# Universal cannabis outcomes from the Climate and Preventure (CAP) study: a cluster randomised controlled trial

**DOI:** 10.1186/s13011-018-0171-4

**Published:** 2018-09-25

**Authors:** Nicola C. Newton, Maree Teesson, Marius Mather, Katrina E. Champion, Emma L. Barrett, Lexine Stapinski, Natacha Carragher, Erin Kelly, Patricia J. Conrod, Tim Slade

**Affiliations:** 1 NHMRC Centre of Research Excellence in Mental Health and Substance Use (CREMS), National Drug and Alcohol Research Centre, University of New South Wales, Sydney, NSW 2052 Australia; 2 Department of Preventive Medicine, Northwestern University Feinberg School of Medicine, Chicago, USA; 3 University of Montreal, Montreal, Canada; 4 CHU Ste Justine, Montreal, Canada

**Keywords:** Cannabis, Prevention, Universal, Combined, School, Selective, Online

## Abstract

**Background:**

The *Climate* and *Preventure* (CAP) study was the first trial to assess and demonstrate the effectiveness of a combined universal and selective approach for preventing alcohol use and related harms among adolescents. The current paper reports universal effects from the CAP study on cannabis-related outcomes over three years.

**Methods:**

A cluster randomized controlled trial was conducted with 2190 students from twenty-six Australian high schools (mean age: 13.3 yrs., SD 0.48). Participants were randomised to one of four conditions; universal prevention for all students (*Climate*); selective prevention for high-risk students (*Preventure*); combined universal and selective prevention (*Climate and Preventure*; CAP); or health education as usual (Control). Participants were assessed at baseline, post intervention (6–9 months post baseline), and at 12-, 24- and 36-months, on measures of cannabis use, knowledge and related harms. This paper compares cannabis-related knowledge, harms and cannabis use in the Control, Climate and CAP groups as specified in the protocol, using multilevel mixed linear models to assess outcomes.

**Results:**

Compared to Control, the Climate and CAP groups showed significantly greater increases in cannabis-related knowledge initially (*p* <  0.001), and had higher knowledge at the 6, 12 and 24-month follow-ups. There was no significant difference between the Climate and CAP groups. While no differences were detected between Control and the CAP and Climate groups on cannabis use or cannabis-related harms, the prevalence of these outcomes was lower than anticipated, possibly limiting power to detect intervention effects. Additional Bayesian analyses exploring confidence in accepting the null hypothesis showed there was insufficient evidence to conclude that the interventions had no effect, or to conclude that they had a meaningfully large effect.

**Conclusions:**

Both the universal Climate and the combined CAP programs were effective in increasing cannabis-related knowledge for up to 2 years. The evidence was inconclusive regarding whether the interventions reduced cannabis use and cannabis-related harms. A longer-term follow-up will ascertain whether the interventions become effective in reducing these outcomes as adolescents transition into early adulthood.

**Trial registration:**

This trial was registered with the Australian New Zealand Clinical Trials Registry (ACTRN12612000026820) on the 6th of January 2012, https://www.anzctr.org.au/Trial/Registration/TrialReview.aspx?id=347906&isReview=true

**Electronic supplementary material:**

The online version of this article (10.1186/s13011-018-0171-4) contains supplementary material, which is available to authorized users.

## Background

Cannabis is the most commonly used illicit drug in most western countries including Australia [1–3]. Despite cannabis being illegal in Australia, an epidemiologic study estimated that 35% of Australians report using cannabis in their life and 10% report use in the past year [2]. Amongst young people aged 14–19 years, 18% reported using cannabis in their lifetime and 15% report using cannabis in the past year. Cannabis use, particularly amongst young people, is associated with considerable burden of disease and social costs [4–7]. Effective prevention is critical to improve the trajectory of young lives and reduce the future development of substance use disorders, and associated mental health epidemiological [8]. Schools offer the ideal location to deliver prevention as it is where students spend a large proportion of their time and educators can reach large audiences at low costs [9]. Despite an increase in the number of school-based programs developed to prevent substance use, many continue to show minimal effects [10–12]. A review specifically focussing on prevention of cannabis use found mixed evidence of benefit, often with small effect sizes, with substantial uncertainty about the relative efficacy of targeted versus universal approaches [13]. It is critical that researchers focus on improving outcomes of school-based substance use prevention programs, particularly in relation to cannabis use, and investigate which treatment approaches are most effective.

The CAP (*Climate* and *Preventure*) study was developed with this aim in mind [14], and was the first trial of a comprehensive model of prevention which combined ‘universal’ (delivered to all students regardless of level of risk), and ‘selective*’* (delivered to specific populations at greatest risk of developing problems) prevention approaches [15]. Specifically, the CAP study aimed to examine the effects of combining two efficacious programs, the universal *Climate Schools* program and the selective personality-targeted *Preventure* program. The programs address substance use from different angles. The *Climate Schools* program focusses on increasing alcohol and cannabis knowledge and changing social norms [16, 17], while the selective *Preventure* program targets four personality profiles and neurocognitive risk factors for substance misuse, namely sensation seeking, impulsivity, anxiety sensitivity and negative thinking, with minimal discussion of substance use [18–20]. Previous research has demonstrated that both programs can individually reduce alcohol consumption, harmful use of alcohol and cannabis use [17, 20, 21], with the universal program also having been shown to improve knowledge about alcohol and cannabis among adolescents [16, 22, 23]. The rationale for combining these approaches was that prevention effects could be maximised by targeting low-risk youth, who may be influenced to take up alcohol due to peer influence and social conformity, as well as youth considered at higher risk, whose underlying personality and neurocognitive vulnerability to psychopathology can lead to early and problematic alcohol and substance misuse [24].

The primary aims of the CAP study were to investigate the effectiveness of universal (*Climate Schools*) and combined (*Climate and Preventure*) prevention in reducing the uptake and harmful use of alcohol and reducing alcohol-related harms, relative to controls (health education as usual). These outcomes have recently been reported with results demonstrating that both universal and combined prevention can delay the uptake of drinking and binge drinking over a two-year period [21, 23]. In the current paper, we report the universal cannabis related outcomes from the cluster randomised controlled trial (RCT). As specified in the protocol, this paper aims to determine whether both universal and combined approaches can be effective in increasing cannabis-related knowledge, decreasing cannabis use, and decreasing cannabis-related harms compared to standard health education as usual. Considering previous efficacy trials of the universal program yielded mild effects on cannabis use outcomes, we hypothesized that the combined approach would be more favourable, where addressing the vulnerabilities of high-risk students may produce additive effects on the prevention of cannabis use and related harms, over and above the effect of a universal intervention, which focuses on cannabis-related knowledge and social norms.

## Methods

### Study design

A cluster RCT was conducted in 26 Australian secondary schools (17 private, 9 public) between 2012 and 2015. Participating schools were randomly assigned to one of four parallel study conditions: (1) ‘Control’, (2) ‘*Climate’*, (3) ‘*Preventure*’, or (4) ‘*Climate and Preventure*’ (CAP). Blocked randomization was conducted by an external researcher using the online program Research Randomiser (https://www.randomizer.org/), allocating schools to each intervention at an equal ratio (1:1:1:1) in blocks of 4. The investigators who recruited schools to participate did not have access to the allocation sequence and were only provided with the next group allocation at the time each school was allocated. Participants, teachers and facilitators were not blinded to intervention status. The CONSORT diagram (see Additional file 1: Fig. S1) summarizes participant flow and retention rates for each trial group over the study period. This study was approved by the University of New South Wales Human Research Ethics Committee, the Sydney Catholic Education Office, and the New South Wales Department of Education and Training. Full details of the study design have been published previously [14, 21]. The current paper reports the cannabis-related outcomes of the RCT, specifically cannabis-related knowledge, cannabis use, and cannabis-related harms, assessing their universal effects among all participants assigned to each intervention. As outlined in the trial protocol [14], the analyses reported in this paper assess universal prevention effects by comparing the Control, Climate and CAP intervention groups (following the protocol, the effects of the standalone *Preventure* intervention were to be assessed in high-risk students only). The positive universal effects of the interventions on preventing alcohol-related outcomes, and the effectiveness of the personality-targeted *Preventure* program on alcohol-related outcomes among ‘high-risk’ students have been reported elsewhere [21, 23, 25].

### Participants and procedure

All Year 8 students (13–14 yrs) attending participating schools in February 2012 were invited to take part in the study. Only students who received parental consent, and consented themselves, were eligible to participate (*n* = 2190). Some schools (*n* = 17) required passive parental consent, while students at other schools (*n* = 9) needed active consent due to ethical requirements. Outcomes were assessed through online or paper questionnaires completed independently by participants. At baseline, all students completed an online self-report survey assessing alcohol and other drug use outcomes, as well as the Substance Use Risk Profile Scale (SURPS), a 23-item questionnaire that assesses personality along four dimensions: sensation seeking, impulsivity, anxiety sensitivity and negative thinking [26, 27]. Students scoring one standard deviation above the school mean on any of the four personality risk subscales were categorized as high-risk. Students with elevated scores on more than one subscale were allocated to the personality group where they deviated most from the mean, according to *z*-scores (see Additional file 1: Figure. S1). Low-risk students were those who did not meet personality risk criteria (i.e., 56.8% of the Year 8 population). All students were invited to complete self-report assessments immediately-post intervention (6 to 9 months post-baseline) and 12, 24 and 36-months after baseline. Study retention was high: 76% (*n* = 1669) completed the post-intervention assessment, 83% (*n* = 1818) completed the 12-month assessment, 79% (*n* = 1732) completed the 24-month assessment and 72% (*n* = 1566) completed the 36-month assessment.

### Interventions

#### Universal intervention (Climate)

Students from schools randomised to the *Climate* condition received the universal *Climate Schools*: *Alcohol and Cannabis course* [28] during health education classes. The *Climate Schools*: *Alcohol and Cannabis course* adopts a social influence approach to prevention and has a harm-minimization goal. The course comprises twelve 40-min lessons aimed at reducing alcohol and cannabis use and related harms. The first six lessons focus on alcohol and were delivered in Term 1, the remaining six lessons focus on alcohol and cannabis and were delivered six months later in Term 3. Each lesson comprised a 20-min online cartoon component completed individually by students, followed by a 20-min group or class activity delivered by the teacher, which reinforces the information in the cartoons and allows interactive communication between students. Teachers were provided with a hard-copy manual and online access to the activities, implementation guidelines, links to the education syllabus, and teacher and student summaries for each lesson. Teachers and students were provided with confidential login details to access the *Climate Schools* website (www.capstudy.org.au). Further details on the content of each lesson are described elsewhere [14].

#### Combined CAP intervention (Climate and Preventure)

The universal *Climate* program was administered to all students in schools randomised to the CAP condition. High-risk students in these schools also received the selective *Preventure* program [29]. *Preventure* is a personality-targeted selective program where students identified as ‘high-risk’ on one of the four SURPS personality subscales are invited to participate. The *Preventure* program comprises two 90-min group sessions, delivered one week apart by a trained facilitator (registered clinical psychologists) and co-facilitator (minimum training: Bachelor of Psychology Honours degree). In the first session, psycho-educational strategies are used to educate students about the target personality style (negative thinking, anxiety sensitivity, impulsivity or sensation seeking) and the associated problematic coping behaviours. Students are encouraged to explore ways of coping with their personality through a goal-setting exercise. Subsequently, students are introduced to the cognitive behavioural model by analyzing a personal experience according to the physical, cognitive, and behavioural responses. In the second session, participants are encouraged to identify and challenge personality-specific cognitive thoughts that lead to problematic behaviours. A total of 81 groups were completed, with an average of 5 students per group. Further details on the *Preventure* program are described elsewhere [14, 18, 25].

#### Program evaluation and implementation fidelity

Following delivery of the interventions, students and teachers in the Climate and CAP groups were asked to complete a questionnaire to provide feedback. Teachers were also asked to complete a logbook indicating which *Climate Schools* lessons and activities they completed with their class (see Additional file 1: Table S1 for fidelity and evaluation data).

#### Health education as usual condition (control)

Schools randomised to the active Control condition received their usual health education classes over the year including lessons on alcohol and other drugs. In Australia, alcohol and other drug education is a mandatory part of the Year 8 health curriculum and all control schools reported delivering drug and alcohol education lessons during this trial. Teachers were asked to provide details about the number and format of these lessons (see Additional file 1: Table S1).

### Measures

Demographic data were obtained (e.g., sex, age, and country of birth). Student responses were linked over time using a unique identification code to ensure confidentiality. Questions about cannabis use were preceded by a note explaining commonly used names for cannabis to ensure participants understood what these questions referred to, i.e. “[s]ome of the more common names for cannabis are marijuana, pot, grass, weed, reefer, joint, MaryJane, cone, spliff, dope, skunk, ganja and hash.”

#### Cannabis-related knowledge

Cannabis-related knowledge was assessed using a 16-item scale that has been used in previous evaluations of the *Climate Schools* programs [16, 23]. The scale assesses knowledge in relation to prevalence of use, risks and information required to minimize harms related to cannabis use. Students were asked to respond to 16 statements (e.g. “*Using cannabis can cause people to feel anxious, depressed (sad), paranoid (suspicious) and panicky.*”), answering ‘true’, ‘false’ or ‘don’t know’ to each. Total scores ranged from 0 to 16, with higher scores indicating higher knowledge. The Cannabis Knowledge scale demonstrated good reliability in the present trial (α = 0.84).

#### Cannabis use

A single item, adapted from the 2010 National Drug Strategy Household Survey (NDSHS) [30], was used to assess cannabis use in the past 6 months (“Have you used cannabis in the past 6 months?” with responses of Yes / No).

#### Cannabis harms

Cannabis-related harms were assessed using a set of questions adapted from the Adolescent Cannabis Problems Questionnaire [31]. Participants were asked whether they had experienced any of the six harms from using cannabis in the past 6 months, for example *“Has your school performance been affected by using cannabis?”.* Possible responses were Never Tried/No/Yes. The Cannabis Harms scale demonstrated acceptable reliability in the present trial (α = 0.80). For analysis of the harms from cannabis outcome, a binary variable was created, coded as 1 for participants experiencing any harms and 0 for participants experiencing no harms.

### Sample size and power calculations

The original sample size calculations for the trial, reported in the published trial protocol [14], were based on methods for sample size calculation in longitudinal cluster RCTs [32] for differences in continuous outcomes (e.g. knowledge). The stated aim was to achieve 80% power to detect a standardized mean difference of 0.3 between groups at the final follow-up, within the high-risk participants who were expected to represent 40% of all participants. The expected effect size of 0.3 was based on previous trials of drug prevention programs [33, 34]. This lead to a desired sample size of 192 high-risk participants in 6 schools per group, and an overall desired sample size of 480 participants in 6 schools per group. Since the full sample is larger than the high-risk subset that sample size calculations were based on, the power to detect effects of the same size is higher.

**Fig. 1:**
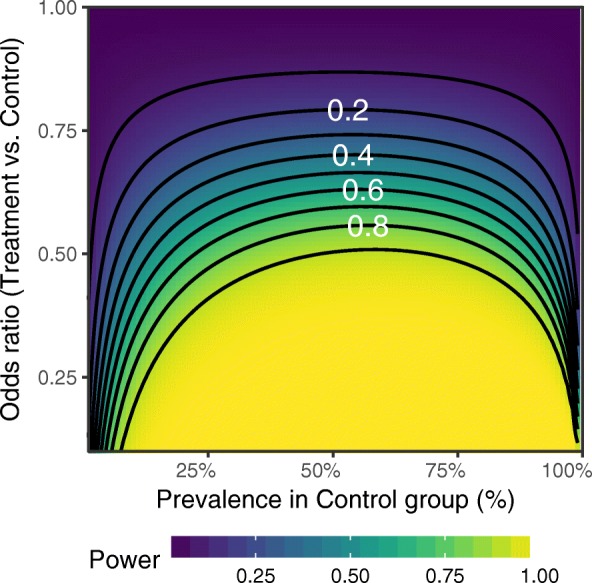
Power to detect differences in binary outcomes as a function of prevalence and effect size. Power was calculated using a method for three-level binary data randomized at the third level [34], using the function: $\upvarphi =\Phi \left\{\frac{\left|{p}_1-{p}_0\right|\sqrt{N_1{N}_2{N}_3/{f}_3}-{\Phi}^{-1}\left(1-\upalpha /2\right)\sqrt{2\overline{p}\left(1-\overline{p}\right)}}{\sqrt{p_0\left(1-{p}_0\right)+{p}_1\left(1-{p}_1\right)}}\right\}$ where *N*_1_ is the size of the level 1 units (repeated observations of participants in the current study); *N*_2_ the size of level 2 units (participants per school); *N*_3_ the number of level 3 units (schools); *p*_1_ and *p*_0_ the prevalence of the outcome in the treatment and control groups respectively; $\overline{p}=\left({p}_0+{p}_1\right)/2$; *f*_3_ = 1 + *N*_1_(*N*_2_ – 1)ρ_2_ + (*N*_1_ – 1)ρ_1_; ρ_1_, ρ_2_ the correlations within level 1 and level 2 units. Power calculations for the trial analyses were performed using *N*_1_ = 5, *N*_2_ = 80, *N*_3_ = 5, ρ_1_ = 0.36, ρ_2_ = 0.03. Contour lines join regions with equal power in increments of 0.1, with the power of some contours labelled in white

For binary outcomes such as cannabis use, calculating power requires taking into account prevalence, which was not considered in the original sample size calculations for the primary outcomes. Power to observe changes in binary outcomes was calculated using a method for power calculation in longitudinal cluster RCTs [35] that was similar to the original sample size calculation approach. Based on effects observed in previous trials of cannabis prevention [36–38], an odds ratio (OR) of 0.7 was selected as the expected effect size. Using the observed prevalence of cannabis use in the control group at the 36-month follow-up (11%), and the observed intraclass correlations for different observations of the same subject (0.36) and participants within the same school (0.03), the power to observe an effect with an odds ratio of 0.7 was calculated as 15%. Full details of the power calculation are given in Fig. 1. As shown in the figure, this low power is partly a result of the low prevalence of cannabis use in the sample, as power for binary outcomes is related to prevalence and decreases when prevalence is below 50%, with substantially lower power to detect outcomes when prevalence is low.

### Statistical analysis

All analyses were carried out based on the intention-to-treat principle, including all available measurements from participants in the intervention groups they were allocated to. Potential differences between participants who completed follow-up assessments and those lost to follow-up were assessed using *χ*^2^ tests or Fisher’s exact test for binary outcomes, and using *t* tests for continuous outcomes.

In the primary analysis, outcomes were modelled using (generalized) multilevel mixed-effects linear models, incorporating random effects at both the school and individual level, with individuals nested in schools. Multilevel mixed modelling approaches are able to model the expected correlation between measurements of the same individual, and between individuals in the same cluster [39], accounting for the non-independence of these observations which would violate the assumptions of standard regression modelling approaches. This is a superior approach for handling missing data, as the models do not require equal numbers of observations per subject, and produce unbiased estimates under both missing completely at random (MCAR) and missing at random (MAR) assumptions when maximum likelihood estimation is used [40]. In order to account for missing data in this study, associations between baseline covariates and non-response were examined, and baseline covariates that predicted non-response were included as additional predictors. Conditional on these predictors of missingness, we assume that the data is MAR, as the available observations for each individual together with the baseline covariates provide information about any missing values.

Regression models incorporated dummy-coded indicators for the Climate and CAP groups with the Control group as the reference level, i.e. coded 1 for the relevant group and 0 otherwise. Time was coded as a continuous variable representing the number of years since baseline, so that the baseline, 6-, 12-, 24- and 36-month measurements were coded as 0, 0.5, 1, 2 and 3 respectively. The effects of the interventions on change in outcomes over time were assessed through Group × Time interaction terms. These models take into account individual differences at baseline, using baseline measurements as the reference point to estimate participant-specific starting points and change over time from these baseline levels.

Growth functions (i.e. the nature of change in outcomes over time) were assessed by testing alternative functions in unconditional models which did not include group predictors. Linear and quadratic time coefficients were successively added to the models, and likelihood-ratio tests were used to determine whether the term improved model fit for that outcome.

Random intercepts and slopes were estimated at the individual level (nested within schools), and random intercepts at the school level. The best-fitting random effects structure for each outcome was tested using likelihood ratio tests, with Akaike information criterion (AIC) statistics used to confirm these comparisons since likelihood ratio tests regarding these parameters are conservative under some conditions [41]. The binary outcomes of cannabis use and cannabis harms were modelled using mixed-effects logistic regression via Stata’s *melogit* command, while the continuous outcome of cannabis knowledge was modelled using the Stata *mixed* command. These analyses were carried out in Stata version 14.2 [42]. In order to interpret the estimated changes, the *margins* and *lincom* commands were used to obtain predicted group means and differences at each measurement occasion.

### Bayesian analyses

Following the primary analyses, it became apparent that we had lower than expected prevalence of cannabis use and harms within our sample. A previous trial of a Climate intervention found baseline levels of cannabis use around 12% [38], and contemporary survey data indicate that recent (past 12 months) cannabis use is evident in 12% of adolescents [43]. In contrast, around 5% of participants in the current trial reported cannabis use at the baseline assessment. Additional Bayesian analyses were therefore conducted after the primary analyses, to investigate and clarify intervention effects where they were non-significant in the primary analyses. Under a conventional null hypothesis significance testing framework, non-significant findings regarding an intervention effect are inherently ambiguous and do not indicate interventions with no true effect [44]. Alternative approaches are required in order to properly distinguish between interventions where there is no true intervention effect and cases where the intervention has a true effect but there is insufficient evidence to identify it (for example due to insufficient power). Bayesian approaches represent a principled alternative, as they allow for conclusions about the evidence in favour of both the null hypothesis and alternative hypotheses. Therefore, they allow for explicitly accepting a null hypothesis of no effect [45]. Bayesian approaches have been recommended for evaluating the effectiveness of interventions, to resolve the ambiguity of non-significant results [46]. In the additional analyses for the current trial, Bayesian multilevel models mirroring the original analyses were applied to each outcome. The region of practical equivalence testing framework [45] was used to assess whether there was sufficient evidence to conclude that the interventions had no effect. Details of the approach, and the results of these analyses are presented in Additional file 2.

## Results

The Control, Climate and CAP intervention groups included 1712 participants, mean age 13.3 years (SD 0.48), 50.5% male. Overall, 86.9% of participants reported they were born in Australia, with 6.8% born in other English-speaking countries and 6.2% in non-English speaking countries.

Baseline characteristics of each intervention group can be seen in Table 1. Some differences between groups appear to be substantial, particularly the proportion of males and females within each group, where the CAP group had a higher proportion of male participants (79.3%) than the Control (33.0%) and Climate (35.9%) groups. This was largely due to the proportion of male-only, female-only and co-educational schools randomly assigned to each group, with a higher proportion of male-only schools assigned to the CAP group (4 out of 6) than the Control (2 out of 7) and Climate (1 out of 6) groups. In line with the CONSORT 2010 guidelines [47] we did not conduct significance tests of baseline differences between intervention groups, but note where the differences are substantial.

**Table 1:** Baseline characteristics of the intervention groups

	Control	Climate	CAP	Missing values
N schools	7	6	6	–
N participants	527	576	609	–
Sex [N male (%)]	174 (33.0)	207 (35.9)	483 (79.3)	0
Age [mean years (SD)]	13.4 (0.43)	13.3 (0.51)	13.3 (0.51)	25
Ever drunk alcohol [N (%)]	77 (14.6)	96 (16.7)	98 (16.1)	4
Ever binge drunk [N (%)]	16 (3.0)	23 (4.0)	27 (4.4)	4

Binge drinking was defined as drinking 5 or more standard alcohol drinks on a single occasion

### Attrition and missing data

Follow-up rates for the four post-baseline surveys are shown in Table 2. Attrition occurred when students failed to remember their account details for completing surveys online, were absent from school on the day of the survey, or failed to provide a correct identification code when completing a paper version of the survey. However, as participants could still complete later surveys after an absence, only 69 participants (4%) completed no follow-up surveys. Attrition analyses were conducted to assess differences in outcomes between participants who were lost to follow-up compared to those who remained. Participants who completed no follow-up assessments did not differ significantly from participants who completed at least one follow-up assessment in terms of the proportion using cannabis at baseline (12.3% vs. 5.8%, ${\upchi}_1^2=3.6$, *p* = 0.058), the proportion of participants experiencing harms from cannabis (4.7% vs. 3.2%, OR = 1.51, 95% CI 0.294 to 4.89, *p* = 0.457, Fisher’s exact test used due to small expected cell counts) or on their mean cannabis knowledge scores (mean score 7.17 [SD 4.22] vs. 6.81 [SD 3.96], t_1689_ =  − 0.71, *p* = 0.478).

**Table 2:** Number of participants completing each follow-up survey

Survey	*N* completed	*N* absent	% completed
Baseline	1712	0	100.0
6 months	1354	358	79.1
12 months	1469	243	85.8
24 months	1395	317	81.5
36 months	1261	451	73.7

As shown in Table 3, most missing cannabis use data in the trial resulted from participants failing to complete surveys, although some data was missing due to participants only partially completing surveys or not completing individual questions. Associations between the different forms of missingness and baseline covariates were examined using chi-squared tests to identify predictors of missingness, as shown in Table 4. Comparing baseline covariates for participants who did not complete any follow-ups after baseline to those who completed at least one showed a significant association between smoking at baseline and not completing follow-up surveys. Sex, baseline cannabis use and baseline smoking were marginally significant in predicting non-response. Comparing baseline covariates for those who completed the cannabis use question at the 36-month follow-up to those who did not showed that sex and drinking at baseline were both significantly associated with not completing the question. Since all of these baseline covariates were at least marginally significant in predicting non-response, they are all included in adjusted analyses in order to properly account for missing data (except baseline cannabis use, which is already accounted for in the model; see Additional file 1: Tables S2a-c).

**Table 3:** Reasons for missing data about cannabis use

Time	Not missing	Did not complete follow-up	Did not complete individual question
Baseline	1689 (98.7%)	0 (0%)	23 (1.3%)
6 months	1249 (73.0%)	358 (20.9%)	105 (6.1%)
12 months	1402 (81.9%)	243 (14.2%)	67 (3.9%)
24 months	1343 (78.4%)	317 (18.5%)	52 (3.1%)
36 months	1152 (67.3%)	451 (26.3%)	109 (6.4%)

**Table 4:** Associations between baseline covariates and missing data for the cannabis use. Associations are presented separately for missing data due to not participating in a given follow-up, or due to not completing individual questions at the 36-month follow-up despite participating in the follow-up

Baseline covariate	Follow-ups completed		χ^2^(1)	*p*
	Completed at least one	Baseline only		
Using cannabis at baseline	94 / 1624 (5.8%)	8 / 57 (12.3%)	3.60	0.058
Sex: male	822 / 1642 (50.1%)	42 / 68 (61.8%)	3.13	0.077
Drinking at baseline	219 / 1640 (13.4%)	15 / 68 (22.1%)	3.48	0.062
Smoking at baseline	117 / 1614 (7.2%)	11 / 64 (17.2%)	7.28	0.007
	Completed question	Did not complete	χ^2^(1)	*p*
Using cannabis at baseline	59 / 1144 (5.2%)	10 / 108 (9.3%)	2.45	0.118
Sex: male	533 / 1151 (46.3%)	96 / 109 (88.1%)	67.8	< 0.001
Drinking at baseline	136 / 1152 (11.8%)	21 / 109 (19.3%)	4.42	0.036
Smoking at baseline	62 / 1138 (5.4%)	9 / 108 (8.3%)	1.04	0.308

#### Multilevel modelling

Table 5 presents the raw means for each outcome by time and intervention group. Estimated coefficients and confidence intervals from the fitted multilevel models are shown in Table 6. Model fit statistics and comparisons are shown in Additional file 1: Tables S3a-f. Comparisons of unconditional models showed that for cannabis use and cannabis-related knowledge, both linear and quadratic change terms were supported, while for cannabis harms only a linear change term was supported. For all outcomes, the best fitting models incorporated random intercepts at the school level, and random intercepts and slopes at the individual level, with independent covariance for cannabis use and cannabis-related knowledge and unstructured for cannabis harms.

**Table 5:** Raw outcome means for each group at each measurement occasion

Outcome	Time	Control	Climate	CAP
Cannabis knowledge score [Mean (SD)]	Baseline	6.85 (3.86)	6.45 (3.97)	7.16 (4.03)
	6 months	7.14 (3.99)	9.95 (4.44)	11.05 (4.07)
	12 months	7.66 (3.98)	9.29 (4.43)	9.99 (4.27)
	24 months	8.33 (4.09)	9.24 (4.14)	9.92 (4.16)
	36 months	9.06 (3.92)	10.04 (3.83)	9.97 (4.44)
Cannabis usage, past 6 months [N (%)]	Baseline	32 (6.1)	32 (5.6)	38 (6.3)
	6 months	37 (8.5)	50 (12.3)	43 (10.5)
	12 months	49 (10.6)	40 (8.5)	51 (10.9)
	24 months	38 (8.8)	40 (9.2)	44 (9.3)
	36 months	42 (10.8)	42 (11.8)	48 (11.8)
Any harms from cannabis, past 6 months [N (%)]	Baseline	14 (2.7)	15 (2.7)	25 (4.2)
	6 months	7 (1.6)	17 (4.2)	26 (6.4)
	12 months	15 (3.2)	13 (2.7)	23 (4.9)
	24 months	9 (2.1)	18 (4.1)	20 (4.2)
	36 months	22 (5.7)	21 (5.9)	25 (6.1)

Cannabis knowledge scores are between 0 and 16, with higher scores reflecting better knowledge about cannabis

**Table 6:** Coefficients from multilevel models assessing change in cannabis knowledge, cannabis use and harms from cannabis

	*Linear change*				*Quadratic change*			
*Outcome*	*b*		*95% CI*	*p*	*b*		*95% CI*	*p*
Cannabis knowledge								
Climate vs Control (ref)	1.97		1.33 to 2.60	< 0.001	−0.62		−0.82 to −0.41	< 0.001
CAP vs Control (ref)	2.25		1.62 to 2.88	< 0.001	−0.80		−1.00 to −0.59	< 0.001
CAP vs Climate (ref)	0.28		− 0.34 to 0.91	0.37	−0.18		−0.38 to 0.03	0.09
*Outcome*	*β*	*Odds ratio*	*OR 95% CI*	*p*	*β*	*Odds ratio*	*OR 95% CI*	*p*
Cannabis use								
Climate vs Control (ref)	−0.12	0.89	0.40 to 1.97	0.77	0.043	1.044	0.81 to 1.35	0.74
CAP vs Control (ref)	−0.08	0.93	0.42 to 2.03	0.85	0.041	1.042	0.81 to 1.35	0.76
CAP vs Climate (ref)	0.04	1.04	0.48 to 2.25	0.91	−0.002	0.998	0.77 to 1.29	0.99
Cannabis harms								
Climate vs Control (ref)	−0.004	0.996	0.68 to 1.45	0.98	–	–	–	–
CAP vs Control (ref)	−0.28	0.759	0.53 to 1.08	0.12	–	–	–	–
CAP vs Climate (ref)	−0.27	0.762	0.54 to 1.07	0.12	–	–	–	–

Health education as usual (Control) was compared to a universal intervention (Climate) and combined universal and selective intervention (CAP). Time was coded in terms of number of years since baseline, so the odds ratios for linear change represent the relative change in odds over one year

b: Unstandardized coefficient on the scale of the original response

β: Coefficient on the logit odds scale

OR 95% CI: 95% confidence interval for the odds ratio

### Cannabis-related knowledge

Based on the model intercepts, the Climate and CAP groups scored higher at baseline than the Control group, but the differences were not significant (Climate: mean difference 0.20, 95% CI -1.10 to 1.51; CAP: mean difference 0.85, 95% CI -0.46 to 2.15), and the Climate and CAP groups did not differ significantly from each other (mean difference 0.64, 95% CI -0.67 to 1.95).

**Fig. 2:**
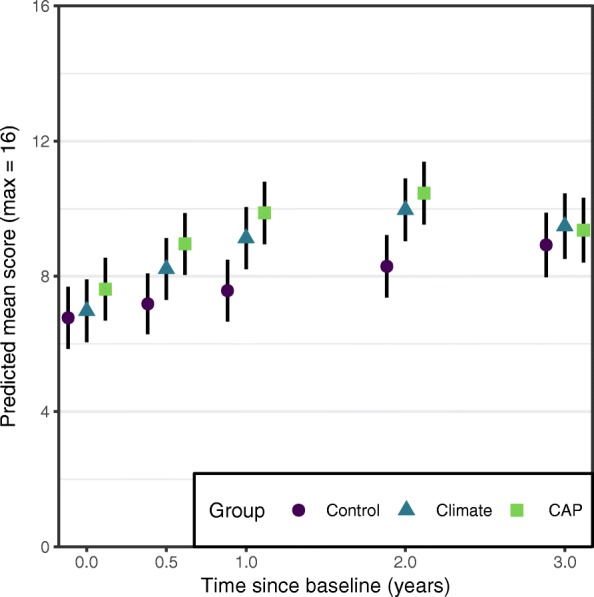
Predicted means of cannabis knowledge at each measurement occasion for each intervention group. Cannabis knowledge scores were on a scale from 0 to 16, with higher scores representing greater knowledge about cannabis. Black lines represent 95% confidence intervals for the predicted mean

As shown in Table 4, both the Climate and CAP groups differed significantly from Control in both their linear and quadratic change. The predicted means from the model, seen in Fig. 2, show that the Climate and CAP groups initially showed a greater increase in knowledge compared to the Control group, but their rate of change slowed over time. Knowledge in the Control group increased gradually over the entire follow-up period, reaching similar levels to the Climate and CAP groups at the 36-month follow-up. The Climate group showed significantly greater change from baseline knowledge than controls at 6 months (mean difference [MD] 0.83, 95% CI 0.56 to 1.10, *d* 0.21, 95% CI 0.14 to 0.28), 12 months (MD 1.35, 95% CI 0.91 to 1.79, *d* 0.34, 95% CI 0.23 to 0.45) and 24 months (MD 1.47, 95% CI 0.92 to 2.01, *d* 0.36, 95% CI 0.23 to 0.49) but not at 36 months (MD 0.35, 95% CI -0.23 to 0.93, *d* 0.09, 95% CI -0.06 to 0.24). The CAP group also showed significantly greater change in knowledge compared to controls at 6 months (MD 0.93, 95% CI 0.66 to 1.19, *d* 0.23, 95% CI 0.17 to 0.30), 12 months (MD 1.45, 95% CI 1.02 to 1.89, *d* 0.37, 95% CI 0.26 to 0.48), 24 months (MD 1.32, 95% CI 0.78 to 1.85, *d* 0.32, 95% CI 0.19 to 0.45), but not at 36 months (MD -0.41, 95% CI -0.97 to 0.16, *d* − 0.10, 95% CI -0.25 to 0.04). The Climate and CAP groups did not show significantly different change from baseline on any occasion.

### Cannabis use

**Fig. 3:**
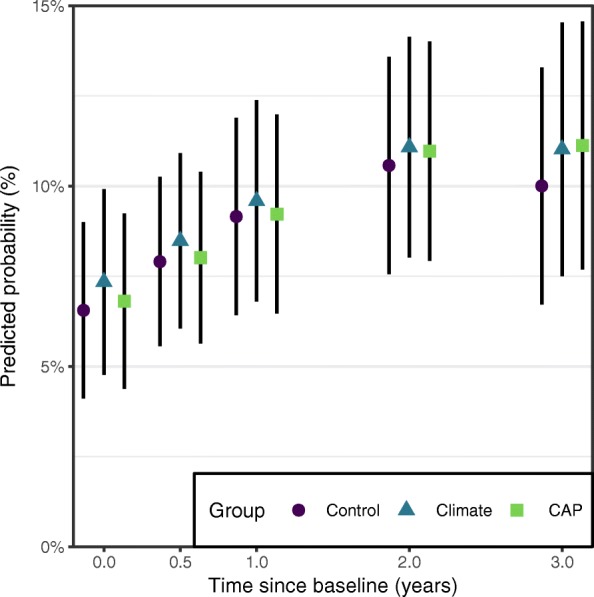
Predicted probabilities of cannabis use at each measurement occasion for each intervention group. A single survey item asked participants whether they had used cannabis in the past 6 months. Black lines represent 95% confidence intervals for the predicted probability

Raw proportions for cannabis usage are shown in Table 3. The Climate and CAP intervention groups were not significantly different in odds of use at baseline compared to the Control group (Climate: OR 1.15, 95% CI 0.62 to 2.12: CAP: OR 1.05, 95% CI 0.57 to 1.93). As seen in Table 4, there were no significant differences between intervention groups for changes in cannabis use over time. Mean predicted probabilities of cannabis use are shown in Fig. 3, showing no significant differences in probability of cannabis use between intervention groups at any follow-up occasion.

### Cannabis harms

**Fig. 4:**
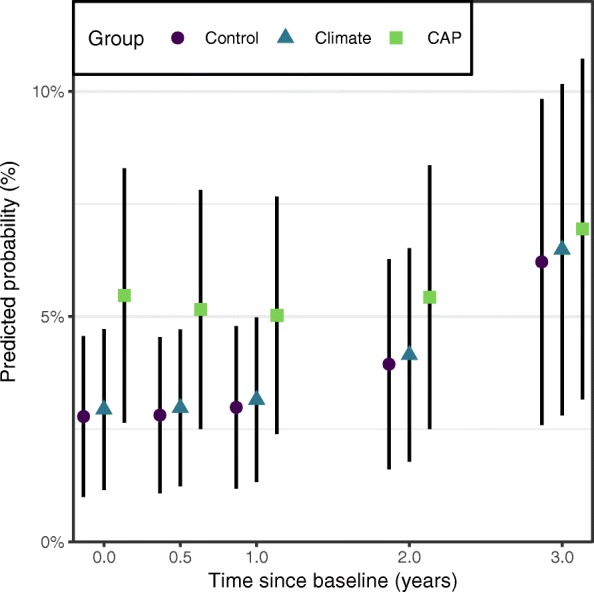
Predicted probabilities of experiencing any harm from cannabis at each measurement occasion. Participants were asked whether they had experienced any of 6 different harms as a result of their cannabis use in the past 6 months. Black lines represent 95% confidence intervals for the predicted probability

The model intercept and group main effects showed non-significant baseline differences between the Climate and CAP groups compared to the Control group (Climate *v.* Control: OR 1.08, 95% CI 0.32 to 3.63; CAP *v.* Control: OR 2.71, 95% CI 0.84 to 8.72), and non-significant differences between Climate and CAP (OR 2.51, 95% CI 0.79 to 7.95). As shown in Table 4, the Climate and CAP groups did not show significantly different change over time in their odds of experiencing harm compared to Control, and did not significantly differ from each other. The mean predicted probabilities from the model, shown in Fig. 4, showed no significant differences between intervention groups at any of the measurement occasions.

#### Bayesian modelling

Results from Bayesian models are presented in (see Additional file 2). For both cannabis use and harms, region of practical equivalence testing showed that there was insufficient evidence to decide in favour of either a meaningful intervention effect or no meaningful difference between the interventions and the control group. For cannabis knowledge, there was evidence of an intervention effect at the 6, 12 and 24-month follow-ups, but at 36 months there was insufficient evidence to conclude in favour of either a meaningful intervention effect or no difference.

## Discussion

Compared to the control group, both the universal *Climate Schools* intervention and the combined CAP intervention were individually successful in increasing cannabis-related knowledge relative to the control group up to the 24-month follow-up, although by the 36 months follow-up the control group had also increased their knowledge to similar levels. This finding replicates previous research showing the *Climate Schools* program improved cannabis-related knowledge [16, 23]. While impacts on knowledge were observed, no significant differences between the control group, and the Climate and CAP groups were observed in change over time in cannabis use and cannabis harms. Given the lower than anticipated prevalence of cannabis use and harms in our sample, Bayesian analyses were conducted to explore confidence in accepting the null hypothesis (e.g., no intervention effect).

These analyses did show some suggestion that the CAP intervention reduced cannabis harms compared to the Control condition, although it was insufficiently strong to reject the null hypothesis of no meaningful difference. Overall, there was not clear evidence to conclude in favour of a meaningful intervention effect or no effect for the Climate and CAP groups compared to Control on cannabis use and harms. Bayesian approaches to assessing evidence are valuable in cases like the current trial, allowing “absence of evidence” to be distinguished from “evidence of absence” and protecting against invalidly concluding that an intervention has no effect from non-significant results [48].

The lack of strong evidence of differences in cannabis use and harms between the intervention and control groups may suggest that the interventions did not focus enough on cannabis to have a strong impact. The *Climate* intervention included an *Alcohol* module as well as a combined *Alcohol and Cannabis* module, meaning that participants received more content related to alcohol than cannabis. Reports from teachers delivering the *Climate* intervention showed that lesson completion rates were high and similar across the *Alcohol* module (90 to 100%) and the *Alcohol and Cannabis Module* (88 to 97%), suggesting that participants received the intervention largely as intended, and therefore the intended amount of cannabis-related material. Given that reductions in alcohol consumption were seen in the trial [21], and the greater amount of time spent on alcohol education during the *Climate* program, it is possible that including more cannabis-specific material, or increasing the time spent on cannabis education, may improve the *Climate* program’s ability to achieve reductions in cannabis use. However, both the Climate Schools and Preventure interventions have previously been shown to reduce cannabis use [16, 36] despite containing limited drug-specific material.

The unexpected low prevalence of cannabis use and harms in our sample may have meant that potential effects of the interventions were difficult to detect. This may reflect changing patterns of cannabis use in Australian adolescents more broadly. Between 11 and 12% of students in the control and intervention groups reported using cannabis at the three-year follow-up, slightly lower than the 14.7% of teenagers of similar age (14–19 years) who report using cannabis in the Australian population [2]. This coincides with the increasing age of cannabis initiation in Australia which has risen from 15.5 years in 2001 to 16.7 years in 2013 [2, 49]. An increased age of initiation may mean that protective effects of preventative interventions will only be seen later, as exposure to cannabis reaches its peak. The interventions’ effects on alcohol grew stronger over time, coinciding with increased levels of alcohol use [21]. Another possibility suggested by the low prevalence of cannabis use observed in this study and the increasing age of initiation in the population is that to be effective, cannabis prevention may need to be delivered later and closer to the typical age of initiation. Interventions delivered in early adolescence may be less effective when prevalence of use is particularly low, as there may be a long delay between the cannabis prevention intervention and participants putting the acquired knowledge and skills into practice upon their first exposure to cannabis. Interventions that closely target the peak period of initiation may therefore be more effective.

Given that cannabis use is associated with alcohol use, the reductions in alcohol use produced by the *Climate and* CAP interventions [21] suggest the potential for concomitant reductions in cannabis use. Previous research conducted with Australian adolescents has shown that cannabis use appears to cluster together with frequent alcohol use [50, 51], and a similar association was present in our sample. A multilevel fixed-effects logistic regression regressing cannabis use against time and recent binge drinking showed that binge drinking was associated with significantly higher odds of cannabis use (OR 6.02, 95% CI 4.22 to 8.60), and this association increased significantly over time (see Additional file 1: Tables S4a-b). To the extent that cannabis use and binge drinking share the same underlying risk factors, there is reason to expect that the reductions in alcohol use and binge drinking observed in the trial will translate into impacts on cannabis use, which may become more apparent as exposure to cannabis increases. However, there may be unique risk factors leading to cannabis use that are not shared with binge drinking, which the interventions do not address sufficiently. Further research may be required to develop interventions that target these factors.

Despite there being no conclusive evidence for differences between groups in cannabis use or related harms in the current study, a longer-term follow-up of the CAP study cohort is warranted to examine if the increases in cannabis-related knowledge we observed in the sample protect against one’s use of cannabis in later years, as exposure to alcohol and other drugs increases. Increased prevalence of cannabis use in the sample would mean greater power to detect intervention effects, as low power may have been a cause of the current analysis producing ambiguous results. In addition, given the success of the interventions in preventing the uptake of alcohol use and binge drinking [21, 23, 25], it is important to examine if these novel interventions have lasting impacts over a critical period as adolescents transition out of school into early adulthood. PROSPER, another school-based intervention program delivered to adolescents, has recently shown long-term reductions in substance use at age 19 [52], demonstrating the potential for early interventions to deliver sustained benefits.

### Strengths and limitations

Strengths of this study include the cluster RCT design allowing for control of contamination effects, the intention to treat analysis, the large sample with high retention, and the use of sophisticated statistical analyses which capture individual differences in trajectories of outcomes and adjust for clustering of data at the school level. The results of this study should be considered in light of potential limitations. Firstly, due to lower than expected cannabis use outcomes in the whole sample, the study might have been underpowered to detect a real effect. Bayesian analyses were reported to guide the reader when interpreting non-significant findings and suggest that there is insufficient evidence to conclude whether there was any effect of Climate on cannabis use and harms, despite clear effects on cannabis-related knowledge.

Another limitation is related to the imbalance in the sex split across the groups, despite the randomised design. Sensitivity analyses adjusting for sex (see Additional file 1: Tables S2a-c) indicated this did not impact substantially on the pattern of results or alter the study conclusions. Secondly, this study relied on self-report data. While this is a potential limitation, our assessment protocol employed all the components required to maximise reliable self-report by young people, and self-reported substance use has been shown to be reliable and valid and is well-accepted in substance use prevention [53, 54]. Whilst retention over 36 months was high, missing data is a limitation common to longitudinal studies, however maximum likelihood estimation was used in our analyses to ensure all available information was used to estimate parameters.

## Conclusions

This study demonstrated that both universal, and combined universal and selective prevention, can be effective in increasing cannabis-related knowledge. Both intervention groups demonstrated effects compared to the control group, and there was no strong evidence to suggest a difference between them. Adding the targeted *Preventure* program to the universal *Climate* intervention therefore did not appear to alter its effects on knowledge. In addition, while no significant differences were revealed for cannabis use outcomes, Bayesian analysis suggested that the evidence was inconclusive about whether this reflected a genuine lack of effect or if the study was under powered to detect a real effect. The longer-term follow-up of the CAP study cohort, which is currently underway, will ascertain whether intervention effects on cannabis use and cannabis-related harms begin to appear as students transition into early adulthood when prevalence of cannabis use may increase.

## Additional files

Additional file 1: Additional figures, tables and supporting information. (DOCX 147 kb)

Additional file 2: Details of Bayesian analyses. Details of the Bayesian methods used, and results of Bayesian analyses. (DOCX 25 kb)

## Abbreviations

AIC: Akaike information criterion
CAP: *Climate* and *Preventure*
MAR: Missing at random
MCAR: Missing completely at random
MD: Mean difference
OR: Odds ratio
RCT: Randomised controlled trial
SURPS: Substance Use Risk Profile Scale
