# Supplementary material for: Universal cannabis outcomes from the Climate and Preventure (CAP) study: a cluster randomised controlled trial
Source: Subst Abuse Treat Prev Policy. 2018 Sep 25;13:34. doi: 10.1186/s13011-018-0171-4 (PMC6157057; doi:10.1186/s13011-018-0171-4)
Supplement: Supplementary file 1 — Additional figures, tables and supporting information. (DOCX 147 kb) [file 13011_2018_171_MOESM1_ESM.docx]

**Supplementary material**

**Figure S1.** CONSORT figure for participant flow in the CAP study.

**Table S1**. Implementation fidelity and program evaluation in the CAP study.

**Table S2a-c**. Results of sensitivity analyses adjusting for sex, drinking at baseline and smoking at baseline.

**Table S3a-f**. Fit statistics and model comparisons for modelling of each outcome in the primary analyses.

**Table S4a-b**. Coefficients and estimated associations from a logistic regression model assessing the relationship between binge drinking and cannabis use.

**Figure S1.** CONSORT figure for participant flow in the CAP study.

190 schools were invited to participate

163 schools declined due to limited time or other commitments

27 schools were recruited (3361 students)

2,608 students gave parental consent

1 school dropped out and 418 students declined participation

## Enrollment

## Baseline

2190 completed baseline survey

## Allocation

609 (27.8%) Climate & Preventure [CAP] (n=6 schools)

340 (55.8%) low risk

269 (44.2%) high risk

478 (21.8%) Preventure†

(n=7 schools)

276 (57.7%) low risk

202 (42.3%) high risk

202 invited to take part in Preventure intervention:

57 (11.9%) scored high on SS

38 (7.9%) scored high on NT

59 (12.3%) scored high on AS

48 (10.0%) scored high on IMP

576 invited to take part in Climate intervention

576 (26.3%) Climate

(n=6 schools)

336 (58.3%) low risk

240 (41.7%) high risk

527 (24.1%) Control

(n=7 schools)

291 (55.2%) low risk

236 (44.8%) high risk

236 not invited to part in interventions:

61 (11.6%) scored high on SS

53 (10.1%) scored high on NT

58 (11.0%) scored high on AS

64 (12.1%) scored high on IMP

609 invited to take part in Climate intervention

269 invited to take part in Preventure intervention:

67 (11.0%) scored high on SS

56 (9.2%) scored high on NT

91 (14.9%) scored high on AS

55 (9.0%) scored high on IMP

6-month follow-up

Assessed**:** 315 (65.9%)

Lost to follow-up: n=163 (34.1%)

6-month follow-up

Assessed**:** 474 (77.8%)

Lost to follow-up: n=135 (22.2%)

## 6mo follow-up*

6-month follow-up

Assessed: 435 (75.5%)

Lost to follow-up: n=141 (24.5%)

6-month follow-up

Assessed: n=445 (84.4%)

Lost to follow-up: n=82 (15.6%)

12-month follow-up

Assessed**:** 517 (84.9%)

Lost to follow-up: n=92 (15.1%)

12-month follow-up

Assessed**:** 349 (73.0%)

Lost to follow-up: n=129 (27.0%)

12-month follow-up

Assessed: n=472 (89.6%)

Lost to follow-up: n=55 (10.4%)

12-month follow-up

Assessed: 480 (83.3%)

Lost to follow-up: n=96 (16.7%)

## 12mo follow-up

24-month follow-up

Assessed**:** 491 (80.6%)

Lost to follow-up: n=118 (19.4%)

(%)

24-month follow-up

Assessed**:** 337 (70.5%)

Lost to follow-up: n=141 (29.5%)

24-month follow-up

Assessed: 454 (78.8%)

Lost to follow-up: n=122 (21.2%)

## 24mo follow-up

24-month follow-up

Assessed: n=450 (85.4%)

Lost to follow-up: n=77 (14.6%)

36-month follow-up

Assessed: n=484 (79.5%)

Lost to follow-up: n=125 (20.5%)

36-month follow-up

Assessed: n=305 (63.8%)

Lost to follow-up: n= 173 (36.2%)

36-month follow-up

Assessed: n= 370 (64.2%)

Lost to follow-up: n= 206 (35.8%)

36-month follow-up

Assessed: n= 407 (77.2%)

Lost to follow-up: n=120 (22.8%)

## 36mo follow-up

Included in analysis (n=609)

Included in analysis (n=478)

Included in analysis (n=576)

Included in analysis (n=527)

## Analysis

*All schools administered the second survey approximately 6 months after baseline, directly after completion of the relevant intervention/s (“immediately post-intervention”)

†Planned comparisons among the full sample of participants did not include the Preventure group, which was intended to be assessed among the high-risk subsample.

**Table S1.** Implementation fidelity and program evaluation in the CAP study.

| **Implementation fidelity of the *Climate Schools* intervention**  A total of 38 teachers from 12 schools returned their completed fidelity logbooks (n=23 from the CAP group, n=11 from the *Climate* group). Completion rates for each of the twelve student lessons ranged from 90% to 100% for the *Alcohol module* and from 88% to 97% for the *Alcohol and Cannabis Module*. Teachers varied widely in terms of which activities, and how many activities they completed with their class for each lesson. All but one teacher (n=37) reported delivering at least one activity for the *Alcohol module* and the *Alcohol and Cannabis* module. The *Climate Schools* course was implemented between February and September, 2012. |
| --- |
| **Implementation fidelity of the *Preventure* interventions**  Of the students randomized to receive the *Preventure* interventions, (n = 1,087), 471 were classified as high-risk on the SURPS and placed into groups (negative thinking = 94; anxiety sensitivity = 150; impulsivity = 103; sensation seeking = 124). These groups were run between March and November 2012. A total of 81 groups (162 sessions) were completed, with an average of five students per group. The majority of students attended the sessions (first session = 90% [n =422]; second session = 84% [n = 394]). |
| **Implementation of standard alcohol and other drugs curriculum (Control group)**  The control schools completed their Personal Development, Health and Physical Education (PDHPE) lessons as usual over the course of the year. The New South Wales PDHPE and the Victorian Health and Physical Education syllabuses mandate that alcohol and other drugs (AOD) education is taught to all Year 8 students, thus all control schools reported implementing some form of universal AOD education throughout the year. Teachers were asked to provide details about the amount and format of any drug education they delivered to their Year 8 students. The number of lessons varied between schools (ranging from two to 10), and the average length of each lesson spent on AOD education was 62 minutes. More than half of teachers (57%) reported using computers or the Internet to teach AOD education topics. The main content areas covered by control schools were: types of drugs, the short and long term effects of AOD, AOD-related laws, decision-making, risk-taking behaviors, patterns of AOD use among young people and the influence of peers and the media. |
| **Student and teacher evaluations of the *Climate Schools* intervention**  A sample of students (n=494) and teachers (n=34) from schools that implemented the *Climate Schools* intervention provided feedback about the course. Overall, evaluation data from both students and teachers was very positive. Nearly all students (93%) agreed that the cartoon stories were an enjoyable way of learning PDHPE theory and that they would like to learn other PDHPE topics in this way (90%). The vast majority of students thought that the information in the cartoons was easy to understand (95%), easy to learn (94%) and easy to remember (94%). Overall, 89% of students reported that they planned to use the information they learnt in the *Climate Schools* program in their own lives. The majority of teachers (88%) indicated that the Climate Schools program was better than other AOD programs, more than three-quarters (77%) indicated that they were likely to recommend the program to others and most (88%) reported that they would be likely to use *Climate Schools* again themselves in the future. |

**Student evaluations of the *Preventure* interventions**

At the completion of the *Preventure* interventions, students were asked to provide anonymous feedback on the relevance, usefulness and acceptability of the program*.* In total, 80% (n=379) of the students completed the student evaluation questionnaire. Almost all students (88%) rated the *Preventure* program as ‘Good’ or ‘Very Good’ overall. The majority of students reported that they found the information in the program helpful (86%) and believed the skills they received in the *Preventure* program would help them to deal more effectively with situations in the future (90%).

**Table S2a-c**. Results of sensitivity analyses adjusting for sex, drinking at baseline and smoking at baseline.

These models use the same fixed effects coefficients and random effects structures as the primary analyses described in the main article, with the addition of the relevant baseline covariates as fixed effects terms.

OR: Odds ratio

CI: 95% confidence interval (for odds ratio)

The model “Adjusting for all” adjusts for sex, baseline drinking and baseline smoking simultaneously.

**Table S2a**. Coefficients from sensitivity analyses for cannabis knowledge, adjusting for sex, drinking at baseline and smoking at baseline.

|  | Adjusting for sex | | | Adjusting for baseline drinking | | | Adjusting for baseline smoking | | | Adjusting for all | | |
| --- | --- | --- | --- | --- | --- | --- | --- | --- | --- | --- | --- | --- |
| Cannabis knowledge | b | CI | p | b | CI | p | b | CI | p | b | CI | p |
| Time | 0.86 | 0.40 to 1.31 | **< 0.001** | 0.85 | 0.40 to 1.30 | **< 0.001** | 0.85 | 0.39 to 1.30 | **< 0.001** | 0.86 | 0.40 to 1.31 | **< 0.001** |
| Time^2^ | 0 | -0.19 to 0.10 | 0.551 | 0 | -0.19 to 0.10 | 0.568 | 0 | -0.19 to 0.10 | 0.565 | -0.046 | -0.19 to 0.10 | 0.542 |
| Group: Climate | 0.2 | -1.10 to 1.49 | 0.768 | 0.18 | -1.11 to 1.47 | 0.783 | 0.19 | -1.10 to 1.49 | 0.771 | 0.17 | -1.10 to 1.45 | 0.791 |
| Group: CAP | 0.87 | -0.44 to 2.18 | 0.193 | 0.81 | -0.48 to 2.10 | 0.220 | 0.79 | -0.51 to 2.08 | 0.233 | 0.78 | -0.51 to 2.07 | 0.234 |
| Group: Climate x Time | 1.95 | 1.32 to 2.59 | **< 0.001** | 1.97 | 1.33 to 2.60 | **< 0.001** | 1.98 | 1.34 to 2.62 | **< 0.001** | 1.97 | 1.33 to 2.61 | **< 0.001** |
| Group: CAP x Time | 2.24 | 1.61 to 2.87 | **< 0.001** | 2.25 | 1.62 to 2.88 | **< 0.001** | 2.28 | 1.64 to 2.91 | **< 0.001** | 2.27 | 1.64 to 2.90 | **< 0.001** |
| Group: Climate x Time^2^ | -0.6 | -0.82 to -0.40 | **< 0.001** | -0.6 | -0.82 to -0.41 | **< 0.001** | -0.6 | -0.82 to -0.41 | **< 0.001** | -0.62 | -0.82 to -0.41 | **< 0.001** |
| Group: CAP x Time^2^ | -0.8 | -1.00 to -0.59 | **< 0.001** | -0.8 | -1.00 to -0.59 | **< 0.001** | -0.80 | -1.01 to -0.60 | **< 0.001** | -0.80 | -1.01 to -0.59 | **< 0.001** |
| Sex: Female | 0.09 | -0.56 to 0.74 | 0.793 | - | - | - | - | - | - | 0.064 | -0.58 to 0.71 | 0.846 |
| Baseline drinking | - | - | - | 0.57 | 0.15 to 0.99 | **0.008** | - | - | - | 0.65 | 0.20 to 1.10 | **0.005** |
| Baseline smoking | - | - | - | - | - | - | 0.11 | -0.45 to 0.66 | 0.704 | -0.20 | -0.79 to 0.40 | 0.516 |
| Intercept | 6.72 | 5.72 to 7.72 | **< 0.001** | 6.72 | 5.81 to 7.62 | **< 0.001** | 6.79 | 5.88 to 7.70 | **< 0.001** | 6.71 | 5.72 to 7.69 | **< 0.001** |
| Observations | 6859 |  |  | 6861 |  |  | 6787 |  |  | 6781 |  |  |

**Table S2b**. Coefficients from sensitivity analyses for cannabis usage, adjusting for sex, drinking at baseline and smoking at baseline.

|  | Adjusting for sex | | | Adjusting for baseline drinking | | | Adjusting for baseline smoking | | | Adjusting for all | | |
| --- | --- | --- | --- | --- | --- | --- | --- | --- | --- | --- | --- | --- |
| Cannabis use | OR | CI | p | OR | CI | p | OR | CI | p | OR | CI | p |
| Time | 1.62 | 0.91 to 2.89 | 0.101 | 1.70 | 0.96 to 2.99 | 0.068 | 1.81 | 1.02 to 3.20 | **0.043** | 1.83 | 1.04 to 3.23 | **0.037** |
| Time^2^ | 0.80 | 0.66 to 0.97 | **0.024** | 0.82 | 0.68 to 0.99 | **0.040** | 0.81 | 0.67 to 0.98 | **0.030** | 0.82 | 0.68 to 0.99 | **0.039** |
| Group: Climate | 1.14 | 0.62 to 2.10 | 0.675 | 1.09 | 0.62 to 1.92 | 0.756 | 1.13 | 0.63 to 2.04 | 0.675 | 1.12 | 0.64 to 1.95 | 0.697 |
| Group: CAP | 1.03 | 0.55 to 1.94 | 0.917 | 0.96 | 0.55 to 1.69 | 0.893 | 0.99 | 0.55 to 1.79 | 0.980 | 0.94 | 0.53 to 1.67 | 0.825 |
| Group: Climate x Time | 0.89 | 0.40 to 1.97 | 0.773 | 0.89 | 0.41 to 1.95 | 0.772 | 0.79 | 0.36 to 1.73 | 0.552 | 0.79 | 0.36 to 1.73 | 0.556 |
| Group: CAP x Time | 0.93 | 0.42 to 2.04 | 0.852 | 0.94 | 0.43 to 2.04 | 0.879 | 0.89 | 0.41 to 1.94 | 0.771 | 0.90 | 0.42 to 1.96 | 0.798 |
| Group: Climate x Time^2^ | 1.04 | 0.80 to 1.35 | 0.749 | 1.04 | 0.81 to 1.34 | 0.752 | 1.08 | 0.84 to 1.40 | 0.535 | 1.08 | 0.84 to 1.39 | 0.545 |
| Group: CAP x Time^2^ | 1.04 | 0.81 to 1.34 | 0.758 | 1.03 | 0.80 to 1.33 | 0.795 | 1.06 | 0.82 to 1.36 | 0.677 | 1.05 | 0.82 to 1.35 | 0.709 |
| Sex: Female | 0.98 | 0.66 to 1.46 | 0.925 | - | - | - | - | - | - | 0.97 | 0.68 to 1.36 | 0.838 |
| Baseline drinking | - | - | - | 4.01 | 3.00 to 5.37 | **< 0.001** | - | - | - | 2.70 | 1.98 to 3.68 | **< 0.001** |
| Baseline smoking | - | - | - | - | - | - | 5.73 | 4.00 to 8.22 | **< 0.001** | 3.48 | 2.38 to 5.08 | **< 0.001** |
| Observations | 6829 |  |  | 6831 |  |  | 6759 |  |  | 6753 |  |  |

**Table S2c**. Coefficients from sensitivity analyses for harms from cannabis, adjusting for sex, drinking at baseline and smoking at baseline.

|  | Adjusting for sex | | | Adjusting for baseline drinking | | | Adjusting for baseline smoking | | | Adjusting for all | | |
| --- | --- | --- | --- | --- | --- | --- | --- | --- | --- | --- | --- | --- |
| Cannabis harms | OR | CI | p | OR | CI | p | OR | CI | p | OR | CI | p |
| Time | 1.64 | 0.99 to 2.70 | 0.053 | 1.48 | 0.91 to 2.40 | 0.112 | 1.68 | 1.07 to 2.64 | **0.024** | 1.57 | 1.00 to 2.47 | 0.051 |
| Group: Climate | 1.16 | 0.42 to 3.22 | 0.777 | 1.07 | 0.33 to 3.48 | 0.914 | 1.06 | 0.35 to 3.20 | 0.912 | 1.15 | 0.48 to 2.73 | 0.759 |
| Group: CAP | 2.03 | 0.74 to 5.55 | 0.168 | 2.46 | 0.79 to 7.71 | 0.122 | 2.15 | 0.74 to 6.23 | 0.157 | 1.48 | 0.63 to 3.46 | 0.366 |
| Group: Climate x Time | 0.99 | 0.68 to 1.45 | 0.971 | 0.98 | 0.67 to 1.43 | 0.911 | 0.98 | 0.67 to 1.44 | 0.924 | 0.97 | 0.66 to 1.43 | 0.877 |
| Group: CAP x Time | 0.76 | 0.53 to 1.08 | 0.123 | 0.77 | 0.54 to 1.09 | 0.137 | 0.76 | 0.53 to 1.09 | 0.137 | 0.77 | 0.53 to 1.10 | 0.148 |
| Sex: Female | 0.43 | 0.21 to 0.85 | **0.015** | - | - | - | - | - | - | 0.36 | 0.20 to 0.64 | **< 0.001** |
| Baseline drinking | - | - | - | 4.70 | 2.82 to 7.82 | **< 0.001** | - | - | - | 2.14 | 1.24 to 3.71 | **0.007** |
| Baseline smoking | - | - | - | - | - | - | 14.54 | 7.91 to 26.72 | **< 0.001** | 10.95 | 5.75 to 20.86 | **< 0.001** |
| Observations | 6825 |  |  | 6827 |  |  | 6761 |  |  | 6755 |  |  |

**Table S3a-f**. Fit statistics and model comparisons for modelling of each outcome in the primary analyses.

Obs: number of observations

LogLik: Model log-likelihood

df: Degrees of freedom

AIC: Akaike information criterion

BIC: Bayesian information criterion

LRT: Likelihood ratio test

**Table S3a**. Comparisons of change over time terms in unconditional models of cannabis knowledge.

| Model | Obs | LogLik | df | AIC | BIC | LRT against previous model | |
| --- | --- | --- | --- | --- | --- | --- | --- |
|  |  |  |  |  |  | χ^2^ | *p* |
| Mean only | 6,865 | -19201.5 | 3 | 38409 | 38429.51 | - | - |
| Linear | 6,865 | -19013.96 | 4 | 38036 | 38063.25 | 375.09 | **< 0.001** |
| Quadratic | 6,865 | -18945.65 | 5 | 37901 | 37935.48 | 136.61 | **< 0.001** |

**Table S3b**. Comparisons of random effects structures for models of cannabis knowledge.

| Model | Obs | LogLik | df | AIC | BIC | LRT against previous model | |
| --- | --- | --- | --- | --- | --- | --- | --- |
|  |  |  |  |  |  | Χ^2^ | *p* |
| Individual intercepts | 6,865 | -18873.55 | 11 | 37769.1 | 37844.3 | - | - |
| School intercepts; Individual intercepts | 6,865 | -18800.24 | 12 | 37624.5 | 37706.5 | 146.6 | **< 0.001** |
| School intercepts; Individual intercepts and slopes (independent)* | 6,865 | -18790.6 | 13 | 37607.2 | 37696.0 | 19.3 | **< 0.001** |
| School intercepts; Individual intercepts and slopes (unstructured) | 6,865 | -18788.75 | 14 | 37605.5 | 37701.2 | 3.71 | 0.054 |

*****Final model selected

**Table S3c**. Comparisons of change over time terms in unconditional models of cannabis use.

| Model | Obs | LogLik | df | AIC | BIC | LRT against previous model | |
| --- | --- | --- | --- | --- | --- | --- | --- |
|  |  |  |  |  |  | χ^2^ | *p* |
| Mean only | 6,835 | -2023.0 | 2 | 4050.0 | 4063.7 | - | - |
| Linear | 6,835 | -2013.9 | 3 | 4033.7 | 4054.2 | 18.29 | **< 0.001** |
| Quadratic | 6,835 | -2011.9 | 4 | 4031.8 | 4059.1 | 3.96 | **0.047** |

**Table S3d**. Comparisons of random effects structures for models of cannabis use.

| Model | Obs | LogLik | df | AIC | BIC | LRT against previous model | |
| --- | --- | --- | --- | --- | --- | --- | --- |
|  |  |  |  |  |  | Χ^2^ | p |
| Individual intercepts | 6,835 | -2011.5 | 10 | 4043.1 | 4111.4 | - | - |
| School intercepts; Individual intercepts | 6,835 | -2007.4 | 11 | 4036.8 | 4111.9 | 8.28 | **0.004** |
| School intercepts; Individual intercepts and slopes (independent)* | 6,835 | -1999.6 | 12 | 4023.1 | 4105.1 | 15.71 | **< 0.001** |
| School intercepts; Individual intercepts and slopes (unstructured) | 6,835 | -1999.5 | 13 | 4024.9 | 4113.7 | 0.16 | 0.689 |

*****Final model selected

**Table S3e**. Comparisons of change over time terms in unconditional models of harm from cannabis.

| Model | Obs | LogLik | df | AIC | BIC | LRT against previous model | |
| --- | --- | --- | --- | --- | --- | --- | --- |
|  |  |  |  |  |  | χ^2^ | *p* |
| Mean only | 6,831 | -1066.4 | 2 | 2136.8 | 2150.4 | - | - |
| Linear | 6,831 | -1058.6 | 3 | 2123.2 | 2143.6 | 15.61 | **< 0.001** |
| Quadratic | 6,831 | -1057.2 | 4 | 2122.3 | 2149.6 | 2.85 | 0.091 |

**Table S3f**. Comparisons of random effects structures for models of harm from cannabis.

| Model | Obs | LogLik | df | AIC | BIC | LRT against previous model | |
| --- | --- | --- | --- | --- | --- | --- | --- |
|  |  |  |  |  |  | χ^2^ | *p* |
| Individual intercepts | 6,831 | -1053.2 | 7 | 2120.5 | 2168.3 | - | - |
| School intercepts; Individual intercepts | 6,831 | -1040.8 | 8 | 2097.7 | 2152.3 | 24.82 | **< 0.001** |
| School intercepts; Individual intercepts and slopes (independent) | 6,831 | -1039.5 | 9 | 2096.9 | 2158.4 | 2.74 | 0.098 |
| School intercepts; Individual intercepts and slopes (unstructured)* | 6,831 | -1037.2 | 10 | 2094.5 | 2162.8 | 4.43 | **0.035** |

*****Final model selected

**Table S4a**. Coefficients from logistic regression for cannabis use regressed on time and current binge drinking.

A mixed-effects logistic regression model with school- and individual-level random intercepts was used to model cannabis use as function of time and binge drinking.

b: Coefficient on logit odds scale

OR: Odds ratio

CI: Confidence interval

| *Cannabis use* | *b* | *OR* | *p* | *OR 95% CI* |
| --- | --- | --- | --- | --- |
| Intercept | -3.05 | 0.05 | **< 0.001** | 0.04 to 0.06 |
| Binge drinking: yes | 1.80 | 6.02 | **< 0.001** | 4.22 to 8.60 |
| Time (years) | -0.14 | 0.87 | **0.01** | 0.79 to 0.97 |
| Binge drinking x Time | 0.25 | 1.28 | **0.006** | 1.07 to 1.53 |

**Table S4b.** Estimated associations (odds ratios) between binge drinking and cannabis use at each assessment time

| *Time* | *OR* | *z* | *p* | *95% CI* |
| --- | --- | --- | --- | --- |
| Baseline | 6.02 | 9.9 | < 0.001 | 4.22 to 8.60 |
| 6 months | 6.82 | 13.0 | < 0.001 | 5.10 to 9.12 |
| 12 months | 7.72 | 16.7 | < 0.001 | 6.08 to 9.81 |
| 24 months | 9.90 | 20.1 | < 0.001 | 7.91 to 12.38 |
| 36 months | 12.69 | 15.3 | < 0.001 | 9.17 to 17.55 |
