# Supplementary material for: Universal cannabis outcomes from the Climate and Preventure (CAP) study: a cluster randomised controlled trial
Source: Subst Abuse Treat Prev Policy. 2018 Sep 25;13:34. doi: 10.1186/s13011-018-0171-4 (PMC6157057; doi:10.1186/s13011-018-0171-4)
Supplement: Supplementary file 2 — Details of Bayesian analyses. Details of the Bayesian methods used, and results of Bayesian analyses. (DOCX 25 kb) [file 13011_2018_171_MOESM2_ESM.docx]

**Appendix A1: Details of Bayesian analyses**

**Methods**

***The region of practical equivalence testing framework.*** Additional Bayesian analyses of the trial outcomes were carried out using the region of practical equivalence testing framework [1] to assess whether effects that were non-significant in the primary analyses represented a genuine lack of intervention effects. In region of practical equivalence testing, a region around the null value of no difference is defined for the intervention effects of interest. This region represents values where there is no meaningful difference between treatments. Analyzing the observed data using a Bayesian model, the posterior distribution of the treatment effect can be tested to determine whether the 95% highest density interval (HDI), representing the most likely values of the effect, falls fully inside or outside of this null region. If the HDI falls entirely outside the null region, we conclude that there is a genuine treatment effect (similar to finding a significant result in null hypothesis significance testing). If the HDI is entirely within the null region, we conclude that there is no practical difference. Alternatively, if the HDI partially overlaps the null region, we conclude there is insufficient evidence to decide whether a genuine effect exists. These conclusions are all valid under the Bayesian framework, unlike in null hypothesis significance testing where it is invalid to infer no difference from a non-significant test of an intervention effect [2].

***Bayesian models.*** Bayesian multilevel models were used to model each outcome – these mirrored the original maximum likelihood analyses by using random effects at the school and individual levels. Regions of practical equivalence were chosen to reflect small differences in outcomes that would not represent meaningful change – half the size of what are considered “small” effect sizes [3, 4]. For the binary outcomes of cannabis use and harms from cannabis, a region of practical equivalence of 0.9 to 1.1 for the odds ratio was used, and for the continuous outcome of cannabis knowledge, a a region of practical equivalence of -0.1 to 0.1 for the effect size *d*.

Models were fit using JAGS version 4.3 [5] using the runjags package for R [6]. All models were fit with 3 chains, each using 20,000 initial “burn-in” samples that were discarded, followed by 20,000 posterior samples, thinning by retaining every 10^th^ sample to reduce autocorrelation. Chains were initialized using maximum likelihood estimates ± 4 standard errors to provide widely dispersed starting points. All models showed good convergence, with Gelman-Rubin statistics < 1.05 for all parameters.

For the binary outcomes of cannabis use and cannabis harm, logistic regression was used, with a Normal(0, 10) prior for the fixed effects coefficients, and a Normal(0, 100) prior for the intercept. For the random effects terms, weakly informative half-Cauchy(2.5) priors were used [7]. For the cannabis knowledge outcome, a Normal(0, 100) prior was used for the intercept, Normal(0, 25) priors for the fixed effect coefficients and a half-Cauchy(0, 25) prior for the standard deviation of scores. Half-Cauchy(25) priors were used for the random effects. Given the range of possible scores for this outcome (0 to 16), these priors are sufficiently wide so as to be weakly informative.

**Results**

Estimated parameters from fitted models are shown in Tables A1a-c. All models showed good convergence, with Gelman-Rubin statistics $\hat{R}<1.05$ for all parameters, and good mixing of all chains. Table A2 shows the posterior HDIs obtained for the intervention effects for each outcome, and the proportion of posterior probability for each of these effects that fell into or outside of the region of practical equivalence. Intervention effects for cannabis use and harms from cannabis were investigated by applying the region of practical equivalence procedure to the odds ratio for each intervention group compared to control at each assessment, controlling for baseline levels.

For both cannabis use and harms, the highest density interval (HDI) for the odds ratio covered regions both inside and outside the region of practical equivalence at all assessment times. Therefore, we conclude there was insufficient evidence to decide in favour of either no meaningful difference or a meaningful intervention effect for Climate and CAP compared to the Control intervention. For cannabis knowledge, the HDI did not overlap the region of practical equivalence for CAP or Climate at the 6, 12 and 24-month follow-ups, showing evidence of an intervention effect, but overlapped at 36 months, showing insufficient evidence to conclude in favour a meaningful intervention effect or no difference.

**Table A1a**. Estimated coefficients *b* (median and 95% credible intervals) from Bayesian regression model for cannabis-related knowledge.

| **Coefficient** | **b (95% credible interval)** |
| --- | --- |
| Intercept | 6.75 (5.70 to 7.78) |
| Group: Climate | 0.23 (-1.30 to 1.72) |
| Group: CAP | 0.87 (-0.62 to 2.34) |
| Time | 0.86 (0.40 to 1.29) |
| Time^2^ | -0.05 (-0.19 to 0.10) |
| Group: Climate x Time | 1.93 (1.32 to 2.59) |
| Group: Climate x Time^2^ | -0.61 (-0.81 to -0.40) |
| Group: CAP x Time | 2.23 (1.58 to 2.84) |
| Group: CAP x Time^2^ | -0.79 (-0.99 to -0.58) |
| **Random effects** | **SD (95% credible interval)** |
| Individual intercepts | 2.44 (2.31 to 2.56) |
| School intercepts | 1.21 (0.77 to 1.81) |

**Table A1b**. Estimated coefficients (*b*) and odds ratios (OR) (median and 95% credible intervals) from Bayesian regression model for cannabis use.

| **Coefficient** | **b (95% credible interval)** | **OR (95% credible interval)** |
| --- | --- | --- |
| Intercept | -3.37 (-3.95 to -2.84) | - |
| Group: Climate | 0.14 (-0.59 to 0.91) | 1.15 (0.55 to 2.48) |
| Group: CAP | 0.04 (-0.69 to 0.73) | 1.04 (0.50 to 2.08) |
| Time | 0.58 (0.04 to 1.16) | 1.78 (1.04 to 3.18) |
| Time^2^ | -0.13 (-0.32 to 0.04) | 0.87 (0.73 to 1.04) |
| Group: Climate x Time | -0.16 (-0.92 to 0.62) | 0.85 (0.40 to 1.86) |
| Group: Climate x Time^2^ | 0.05 (-0.20 to 0.29) | 1.06 (0.81 to 1.34) |
| Group: CAP x Time | -0.09 (-0.87 to 0.69) | 0.91 (0.42 to 2.00) |
| Group: CAP x Time^2^ | 0.04 (-0.21 to 0.30) | 1.04 (0.81 to 1.35) |
| **Random effects** | **SD (95% credible interval)** |  |
| Individual intercepts | 1.29 (1.11 to 1.47) |  |
| School intercepts | 0.38 (0.16 to 0.67) |  |

**Table A1c**. Estimated coefficients (*b*) and odds ratios (OR) (median and 95% credible intervals) from Bayesian regression model for cannabis harms.

| **Coefficient** | **b (95% credible interval)** | **OR (95% credible interval)** |
| --- | --- | --- |
| Intercept | -5.38 (-6.44 to -4.41) | - |
| Group: Climate | 0.10 (-1.18 to 1.32) | 1.10 (0.31 to 3.76) |
| Group: CAP | 0.91 (-0.32 to 2.08) | 2.49 (0.73 to 8.03) |
| Time | 0.39 (0.14 to 0.64) | 1.48 (1.15 to 1.89) |
| Group: Climate x Time | -0.02 (-0.36 to 0.30) | 0.98 (0.70 to 1.35) |
| Group: CAP x Time | -0.27 (-0.58 to 0.05) | 0.76 (0.56 to 1.05) |
| **Random effects** | **SD (95% credible interval)** |  |
| Individual intercepts | 1.87 (1.57 to 2.22) |  |
| School intercepts | 0.82 (0.45 to 1.33) |  |

**Table A2**: Estimated odds ratio (OR) and effect size (*d*) from Bayesian analyses of cannabis outcomes.

| *Knowledge* | *Time* | *d [median (95% HDI)]* | *p(ROPE) (%)* | *p(d < -0.1) (%)* | *p(d > 0.1) (%)* |
| --- | --- | --- | --- | --- | --- |
| Climate | 6m | 0.21 (0.14 to 0.27) | 0.2 | < 0.1 | 99.8 |
|  | 12m | 0.34 (0.22 to 0.44) | < 0.1 | < 0.1 | > 99.9 |
|  | 24m | 0.36 (0.22 to 0.48) | < 0.1 | < 0.1 | > 99.9 |
|  | 36m | 0.09 (-0.05 to 0.23) | 53.9 | 0.3 | 45.8 |
| CAP | 6m | 0.23 (0.17 to 0.30) | < 0.1 | < 0.1 | > 99.9 |
|  | 12m | 0.36 (0.26 to 0.47) | < 0.1 | < 0.1 | > 99.9 |
|  | 24m | 0.32 (0.20 to 0.44) | < 0.1 | < 0.1 | > 99.9 |
|  | 36m | -0.10 (-0.23 to 0.04) | 49.6 | 50.2 | 0.1 |
| *Harm* | *Time* | *OR [median (95% HDI)]* | *p(ROPE)* ***(%)*** | *p(OR < 0.9)* ***(%)*** | *p(OR > 1.1)* ***(%)*** |
| Climate | 6m | 0.99 (0.84 to 1.16) | 75.7 | 13.2 | 11.1 |
|  | 12m | 0.98 (0.70 to 1.35) | 43.8 | 31.1 | 25.1 |
|  | 24m | 0.96 (0.45 to 1.75) | 22.8 | 42.5 | 34.7 |
|  | 36m | 0.94 (0.22 to 2.20) | 14.9 | 46.6 | 38.5 |
| CAP | 6m | 0.87 (0.74 to 1.02) | 35.1 | 64.6 | 0.3 |
|  | 12m | 0.76 (0.54 to 1.02) | 13.8 | 85.1 | 1.1 |
|  | 24m | 0.58 (0.27 to 1.01) | 6.1 | 91.3 | 2.6 |
|  | 36m* | 0.45 (0.12 to 0.99) | 4 | 92.9 | 3.1 |
| *Use* | *Time* | *OR [median (95% HDI)]* | *p(ROPE)* ***(%)*** | *p(OR < 0.9)* ***(%)*** | *p(OR > 1.1)* ***(%)*** |
| Climate | 6m | 0.94 (0.66 to 1.28) | 42.9 | 40.8 | 16.4 |
|  | 12m | 0.90 (0.47 to 1.46) | 26.6 | 49.9 | 23.5 |
|  | 24m | 0.91 (0.37 to 1.56) | 22.2 | 49.3 | 28.5 |
|  | 36m | 1.01 (0.48 to 1.78) | 24.5 | 35.9 | 39.6 |
| CAP | 6m | 0.97 (0.68 to 1.33) | 44.6 | 33.5 | 21.9 |
|  | 12m | 0.95 (0.52 to 1.59) | 27.8 | 41.6 | 30.6 |
|  | 24m | 0.99 (0.44 to 1.76) | 23.1 | 39.4 | 37.5 |
|  | 36m | 1.10 (0.52 to 1.90) | 23.7 | 25.7 | 50.6 |

Effects are reported as posterior median and 95% highest density interval (HDI). Cannabis use, cannabis-related harm and cannabis knowledge in the Climate and CAP groups were compared to outcomes in the Control group. Estimates are based on estimated change in each outcome, controlling for baseline levels. *p*(ROPE) is the posterior probability that the estimate fell in the region of practical equivalence for each outcome where there is no meaningful difference between the treatments. Posterior probabilities are also given for parameter values outside of this region. A higher posterior probability in the region of practical equivalence represents stronger evidence that there is no practical difference between the intervention group and control. For example, the CAP intervention was associated with a 55% reduction in likelihood of reporting cannabis related harms at 36-month follow-up relative to control group; there is a 92.9 % posterior probability that this statistic represents a real decrease at the population level, a 3.1% posterior probability of an iatrogenic effect, and a 4% probability that there is no difference between groups.

**References**

1. Kruschke JK: **Bayesian Assessment of Null Values Via Parameter Estimation and Model Comparison.** *Perspect Psychol Sci* 2011, **6:**299-312.

2. Altman DG, Bland JM: **Absence of evidence is not evidence of absence.** *BMJ* 1995, **311:**485.

3. Cohen J: **A Power Primer.** *Psychological Bulletin* 1992, **112:**155-159.

4. Olivier J, May WL, Bell ML: **Relative effect sizes for measures of risk.** *Communications in Statistics-Theory and Methods* 2017, **46:**6774-6781.

5. Plummer M: **JAGS: A Program for Analysis of Bayesian Graphical Models using Gibbs Sampling.** 2003.

6. Denwood MJ: **runjags: An R Package Providing Interface Utilities, Model Templates, Parallel Computing Methods and Additional Distributions for MCMC Models in JAGS.** *2016* 2016, **71:**25.

7. Gelman A: **Prior distributions for variance parameters in hierarchical models(Comment on an Article by Browne and Draper).** *Bayesian Analysis* 2006, **1:**515-533.
